# Molecular Mechanisms of Lignans in Lowering Blood Pressure and Anti-Obesity Effects: A Review

**DOI:** 10.3390/foods15020336

**Published:** 2026-01-16

**Authors:** Gitishree Das, Sandra Gonçalves, José Basilio Heredia, Nayely Leyva-López, Anabela Romano, Spiros Paramithiotis, Han-Seung Shin, Jayanta Kumar Patra

**Affiliations:** 1 Department of Food Science and Biotechnology, College of Life Science and Biotechnology, Dongguk University-Seoul, Goyang-si 10326, Republic of Korea; 2 MED—Mediterranean Institute for Agriculture, Environment and Development & CHANGE—Global Change and Sustainability Institute, Faculdade de Ciências e Tecnologia, Universidade do Algarve, Campus de Gambelas, 8005-139 Faro, Portugal; 3 Centro de Investigación en Alimentación y Desarrollo, A.C. Subsede Culiacán, Carretera a Eldorado Km. 5.5, Col. Campo El Diez, Culiacán 80110, Sinaloa, Mexico; 4 Posdoc SECIHTI—Centro de Investigación en Alimentación y Desarrollo, A.C. Unidad Culiacán, Carretera a Eldorado Km. 5.5, Col. Campo El Diez, Culiacán 80110, Sinaloa, Mexico; 5 Department of Biological Applications and Technology, University of Ioannina, 45110 Ioannina, Greece; paramithiotis@uoi.gr

**Keywords:** lignans, dietary polyphenols, phytochemicals, secoisolariciresinol diglucoside, molecular mechanism

## Abstract

Lignans are naturally occurring compounds found in a wide variety of plant species, including flaxseed, soybean, pumpkin seed, broccoli, sesame seed, and some berries. Lignans have been used for centuries in both food and traditional herbal medicine. Recently, numerous new lignans and lignan derivatives with diverse biological properties have been identified. Lignans are considered promising for human health due to their hydrogen-donating antioxidant activity together with their ability to complex divalent transition metal cations. They have demonstrated beneficial effects for cardiovascular disease, as well as in maintaining blood glucose levels, supporting cardiac health, promoting anti-obesity effects, decreasing the risk of renal diseases, enhancing brain function, improving skin and gut health, among others. This review explores the biosynthesis and biological effects of lignans, with a particular focus on their antihypertensive and anti-obesity properties, as well as the molecular mechanisms involved. It also highlights recent advances in sustainable lignan extraction techniques that are suitable for human use. The mechanisms underlying these bioactivities are thought to involve hormonal metabolism and availability, antioxidant action, modulation of angiogenesis, and more. However, further research is needed to fully elucidate the molecular pathways through which lignans exert their therapeutic effects. Overall, lignans from various plant sources hold significant potential for application in functional foods, dietary supplements, and pharmaceutical products aimed at preventing and managing a range of health conditions, including hypertension and obesity.

## 1. Introduction

**Figure 1:**
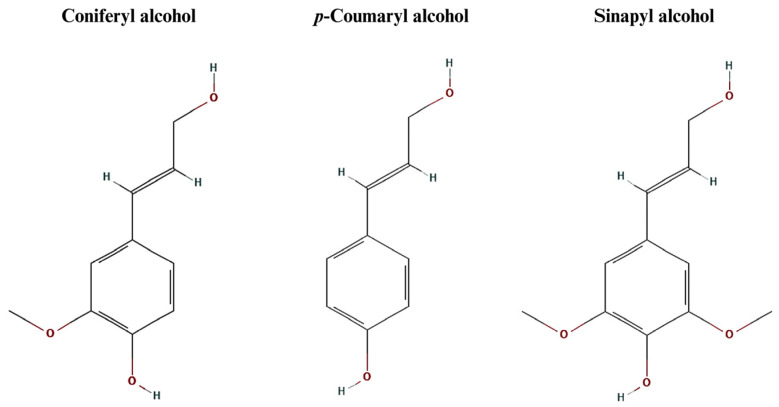
Chemical structures of the most common monolignols that form lignans. Source [5,6,7].

Lignans are a diverse class of naturally occurring polyphenolic compounds predominantly found in plants. Structurally, they consist of two phenylpropanoid units (C6-C3) linked by their central carbon atoms, forming a dimeric structure [1]. This unique configuration distinguishes them from other plant phenolics and contributes to their biological activities. The fundamental framework of lignans consists of two phenylpropanoid units linked primarily through the β (carbon 8) positions, forming a C8–C8’ bond. This linkage results in a 2,3-dibenzylbutane skeleton, which serves as the backbone for various lignan structures [2]. The most common phenylpropanoid units that form lignans, also known as monolignol units, are coniferyl, *p*-coumaryl, and sinapyl alcohols (Figure 1). The specific pattern of linkage and the degree of oxidation or reduction at key positions lead to the vast diversity observed within this class of compounds [3]. Thus, there are eight subgroups of lignans: furan, furofuran, dibenzylbutane, dibenzylbutyrolactol, dibenzylbutyrolactones, dibenzocyclooctadienes, aryltetralin, and arylnaphthalene [4].

Lignans are mainly found in several plant-based foods, especially in seeds, whole grains, vegetables, and fruits, but they are also present in beverages, such as wine, tea, and coffee [8]. Certain oilseeds, particularly flaxseeds (*Linum usitatissimum* L.) and sesame (*Sesamum indicum* L.) seeds, are recognized for their exceptionally high lignan content. Flaxseeds present a total lignan content of around 9.18–125.97 mg/100 g dry weight and are a primary source of the lignan secoisolariciresinol [9]. Recently, it has been reported that the total content of lignan in 40 varieties of flaxseeds varies from 218.54 ± 1.71 to 2505.76 ± 14.75 mg/100 g dry weight [10]. Regarding sesame seeds of different varieties, it is reported that this food presents a total lignan content ranging from 5.22 ± 0.02 to 12.68 ± 0.18 mg/g [11]. The sesame seeds contain sesamin, sesamolin, and sesaminol as the most abundant lignans [12]. Smeds et al. [13], reported the total lignan content of several seeds and berries. Among the seeds, flaxseeds and sesame seeds were the main sources of lignans, while hulled hemp (0.297 mg/100 g) and pumpkin (0.277 mg/100 g) seeds showed lower lignan content. Rye bran (4.499 mg/100 g) had a higher lignan concentration than wheat (3.084 mg/100 g) and oat (1.464 mg/100 g) brans. Regarding the berries, cloudberries (14.775 mg/100 g) and blackberries (9.995 mg/100 g) presented higher values of lignan concentration. In contrast, the berries with the lowest lignan content were blackcurrants (1.007 mg/100 g) and red gooseberries (1.276 mg/100 g). It is important to mention that lignan content might vary depending on the variety and extraction conditions [14,15].

**Figure 2:**
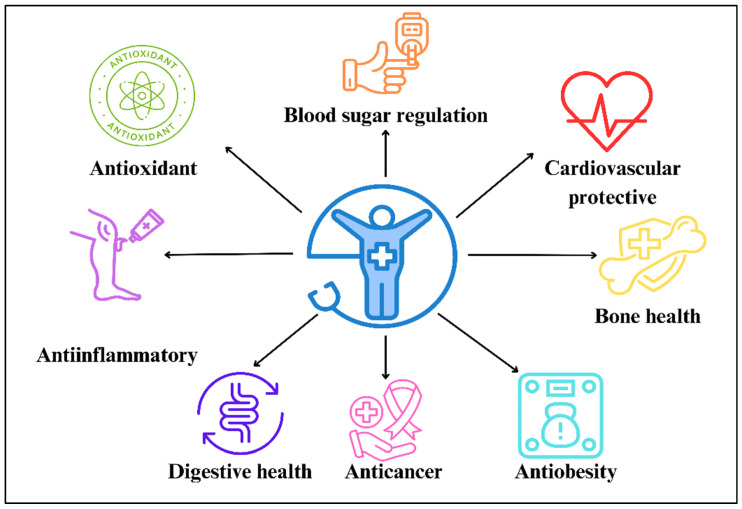
The main health benefits attributed to lignan food intake.

Many vitamins and phytochemicals present in natural sources have high antioxidant properties that reduce oxidative stress and cellular damage [16]. The unique chemical structure of lignans allows them to act as phytoestrogens and antioxidants, contributing to a range of health benefits such as anti-inflammatory, anticancer, alleviating menopausal symptoms, reducing the risk of type 2 diabetes, and protecting cardiovascular health, among others [17] (Figure 2). For instance, lignans have been tested against breast, ovarian, lung, and colorectal cancer, among others [18,19,20,21]. Additionally, human lignan intake has been associated with reducing the homeostatic model assessment for insulin resistance (HOMA-IR) values related to preventing obesity and diabetes [22]. Furthermore, male Wistar rats (150–170 g) were fed with a high-calorie diet and treated with 20 mg/kg/ day of flaxseed lignan extract containing secoisolariciresinol diglucoside (SDG) for 12 weeks. At the end of the experiment, rats treated with the lignan extract showed lower weight, total cholesterol, and total triglycerides, and a higher level of LDL-cholesterol than the rats fed only with the high-calorie diet. Furthermore, the atherogenic coefficient, atherogenic index, and cardiac risk ratio were significantly reduced when rats were fed the lignan extract, which led the authors to conclude that the lignan flaxseed extract exerts cardiac- and vascular-protective potentials [23].

As mentioned, recent research emphasizes the health-promoting effects of lignans due to their bioactive properties. Consequently, consuming foods rich in lignans may serve as an effective strategy to support the prevention of chronic diseases, including specific types of cancers, diabetes, and cardiovascular conditions. In this context, the present review comprehensively examines the molecular mechanisms and health benefits of lignans, particularly focusing on their roles in reducing blood pressure and combating obesity by integrating evidence from lignan biosynthesis pathways, in vitro and in vivo models, clinical trials, and recent advances in extraction and standardization technologies. Particularly, we address the following research question: What are the most strongly supported molecular mechanisms and signaling pathways through which lignans lower blood pressure and exert anti-obesity effects, and how do lignan source/matrix and gut biotransformation into enterolignans influence the magnitude and consistency of these outcomes across biosynthetic evidence, in vitro and in vivo models, and clinical trials.

## 2. Methodology

In the current review, a literature search and selection methodology for articles and patents on lignans from different plant sources was carried out using the most popular bibliographic and patent databases and search engines such as Scopus, Pubmed, Science Direct, and Google Scholar, using the keywords such as flaxseed lignans, molecular mechanism of lignans, dietary polyphenols, phytochemicals, secoisolariciresinol diglucoside, etc. This review provides an overview of the main effects of lignans in lowering blood pressure and anti-obesity–related effects by integrating data from both dietary lignan-rich foods/products and isolated compounds from different sources. This data revision is preceded by a summary of key steps in lignan biosynthesis and complemented with a revision of the main extraction procedures used to extract these compounds. We retrieved the recently published studies on lignan through the Web of Science to give an inclusive understanding of the research developments of lignan, especially understanding its effects and molecular mechanism for lowering blood pressure and anti-obesity effects. Results showed that lignan from various plant sources has been widely applied to many industries (ex., food, pharmaceutical, and cosmetics) with potential applications as an antioxidant, anti-cardiovascular disease, anti-cancer, in osteoporosis and diabetes prevention.

## 3. Biosynthesis of Lignans

**Figure 3:**
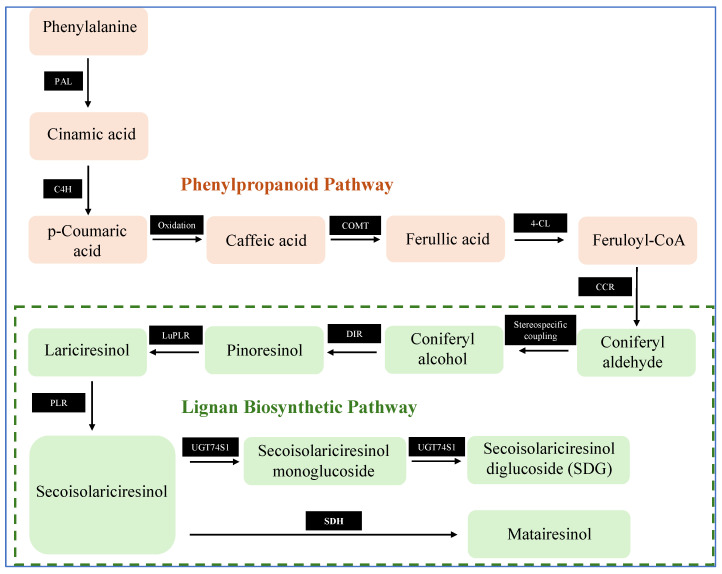
Biosynthesis of lignans. PAL: phenylalanine ammonia-lyase; C4H: cinnamate 4-hydroxylase; COMT: caffeic acid *O*-methyltransferase; 4-CL: 4-coumarate-CoA ligase; CCR: cinnamoyl-CoA reductase; DIR: dirigent proteins; LuPLR: *Linum usitatissimum* pinoresinol lariciresinol reductase; PLR: Pinoresinol-lariciresinol reductase; UGT74S1: Uridine glycosyl transferase 74S1; SDH: secoisolariciresinol dehydrogenase.

Lignans are naturally occurring phenolic compounds found in various parts of plants, such as seeds, roots, stems, leaves, and fruits. Among dietary sources, flaxseed, sesame, and *Brassica* vegetables are the richest in lignans. In flaxseed, the predominant lignans are secoisolariciresinol (SECO) and matairesinol (MAT); pinoresinol (PINO) and 1-ariciresinol (LARI) are the main lignans in Brassica species, while PINO is also abundant in sesame [24]. The biosynthesis of lignans originates from coniferyl alcohol, a product of the phenylpropanoid pathway. This pathway begins from the aromatic amino acid phenylalanine (Phe), which undergoes a series of enzymatic reactions in the cytoplasm [25,26] (Figure 3).

Initially, phenylalanine ammonia-lyase (PAL) catalyzes the deamination of Phe to form cinnamic acid, which is then hydroxylated by cinnamate 4-hydroxylase (C4H) to produce p-coumaric acid. Successive enzymatic steps transform p-coumaric acid into caffeic acid, which is methylated by caffeic acid O-methyltransferase (COMT) to generate ferulic acid. This compound is then transformed into feruloyl-CoA by 4-coumarate-CoA ligase (4CL), and subsequently reduced to coniferyl aldehyde by cinnamoyl-CoA reductase (CCR). The final step involves the reduction in coniferyl aldehyde by cinnamyl-alcohol dehydrogenase and sinapyl alcohol dehydrogenase, yielding coniferyl alcohol, the key precursor of lignans.

The key step in lignan biosynthesis is the stereoselective radical dimerization of coniferyl alcohol, mediated by dirigent proteins (DIR). DIR guides the coupling of two coniferyl alcohol radicals, ensuring specific stereochemistry in the production of PINO [27]. From PINO, two major biosynthetic branches emerge, each leading to different structural types of lignans via specific enzymatic transformations [26,28]. Reduction Pathway—the furan ring in PINO is reduced to form dibenzylbutane lignans, like LARI and SECO [29,30]. Furofuran Pathway—the furan structure is retained and further modified to produce methylenedioxy-bridged furanone lignans, such as sesamin (SES), which is synthesized primarily in *Sesamum* species via the addition of two methylenedioxy bridges [25,31].

The biosynthesis of dibenzylbutane lignans like SECO involves three key enzymatic steps: stereoselective coupling guided by DIR, reduction mediated by PINO–LARI reductases (PLRs), and finally glycosylation catalyzed by uridine glycosyltransferases (UGTs). In flax, the biosynthetic pathway proceeds as follows: PINO is first reduced to LARI, then to SECO, both steps catalyzed by a bi-functional NADPH-dependent PLR [32,33]. DIR genes encode both (−) and (+) stereoisomers of PINO. In flax, the DIR gene responsible for producing (−) PINO is expressed early during seed coat development and ultimately leads to the formation of (+) (SECO). This is subsequently glycosylated by UGT74S1, forming secoisolariciresinol diglucoside (SDG)—a stable, water-insoluble lignan glycoside uniquely present in seeds [34]. UGT enzymes, members of the glycosyltransferases superfamily, are responsible for modifying plant secondary metabolites and improving their stability, solubility, and chemical diversity [35]. Conversely, DIR encoding (+) PINO produces (−) SECO in the aerial plant tissues, where SDG is typically absent [36]. Additionally, SECO can be converted into MAT via the action of secoisolariciresinol dehydrogenase (SDH) [37,38]. MAT acts as a key intermediate in the biosynthetic pathway leading to the pharmacologically significant lignan podophyllotoxin, known for its anticancer properties [39].

## 4. Molecular Mechanisms of Lignans in Lowering Blood Pressure (Antihypertensive) and in Anti-Obesity Effects

### 4.1. Antihypertensive Effects

Lignans, a class of polyphenolic compounds, have long been recognized for their broad range of health benefits, namely antioxidant, anticancer, anti-inflammatory, antidiabetic, estrogenic, anti-estrogenic, anti-obesity, and antihypertensive activities [28].

Hypertension, a highly prevalent condition affecting over one billion individuals worldwide, is a main risk factor for numerous cardiovascular complications and remains a leading cause of global mortality [40]. Although pharmacological therapies are the cornerstone of antihypertensive treatment, dietary and lifestyle interventions are also fundamental to blood pressure management. Among dietary components, lignans have attracted growing attention due to their potential antihypertensive effects. Lignans are abundant in flax and sesame seeds, among other plant sources. However, these compounds are not directly metabolized by digestive enzymes in the human gastrointestinal tract [41]. Instead, gut microbiota converts dietary lignans into bioactive enterolignans, primarily enterodiol (END) and enterolactone (ENL), which are considered the main physiologically active forms [26]. These metabolites are absorbed systemically and contribute to the biological effects of lignans [42]. Consequently, the gut microbiota significantly influences the bioavailability and biological activity of lignans, highlighting the need to better understand the mechanisms underlying lignan–microbiota interactions. Supporting this, a recent population-based study in the United States reported that higher circulating levels of enterolactone—used as a marker of gut microbial activity—were inversely associated with the risk of hypertension [43].

Flaxseed is among the richest dietary sources of lignans and has been widely investigated for its antihypertensive effects, particularly in animal models, and the main achievements are compiled in Table 1. Sawant and Bodhankar [44], demonstrated that oral administration of a flax lignan concentrate (200, 400, and 800 mg/kg) to Wistar rats with deoxycorticosterone acetate-salt-induced hypertension reduced blood pressure. The highest dose exerted its effects through modulation of endogenous antioxidant enzymes, suppression of reactive oxygen species, and potential antagonism of the renin-angiotensin–aldosterone system. Similarly, Park and Velasquez [45], demonstrated that dietary supplementation with SDG-enriched flaxseed powder (0.02%) significantly reduced blood pressure in rats fed a high-fat, high-fructose diet. Prasad [46], who found that SDG reduced blood pressure in Dawley male rats by inhibiting angiotensin-converting enzyme (ACE), further elucidated the underlying mechanism. Notably, SDG is not bioavailable in its original form; instead, it is converted into SECO by gut microbiota and subsequently metabolized into END and ENL [47].

In addition to dietary sources, several medicinal plants are rich in lignans with demonstrated antihypertensive activity. For instance, phillygenin, a lignan compound, significantly lowered SBP and DBP in hypertensive rats following oral administration at doses ranging from 2.5 to 10 mg/kg. Mechanistic studies revealed that phillygenin directly binds to PLCβ3 protein, thereby inhibiting the generation of inositol trisphosphate (IP3) and intracellular Ca^2+^ release, ultimately promoting vasodilation [49]. Another notable example is gomisin J, a lignan isolated from *Schisandra chinensis*, a medicinal plant widely used in East Asian traditional medicine. This compound attenuated the angiotensin II-induced hypertension in mice by enhancing nitric oxide bioavailability [48].

The clinical evidence of the antihypertensive effects of lignans in humans is reported in a few studies. The effect of daily ingestion of flaxseed on systolic (SBP) and diastolic blood pressure (DBP) was investigated in a randomized trial with peripheral artery disease patients (110 in total) [50]. Patients ingested foods containing 30 g of milled flaxseed or a placebo daily for 6 months. It was observed that dietary lignan-rich foods lowered both SBP and DBP in humans, particularly in those with elevated baseline BP. Several systematic reviews and meta-analyses of controlled trials have concluded that flaxseed and flaxseed-derived products may lead to modest reductions in blood pressure, particularly diastolic blood pressure, especially when consumed in whole-seed form and over 12 weeks [51,52,53]. Sesame seeds, another important dietary source of lignans, have also been associated with blood pressure reduction. A meta-analysis by Khosravi-Boroujeni et al. [54], confirmed the antihypertensive potential of sesame intake. Sesame contains several bioactive lignans, such as sesamin, sesamolin, sesamol, and episesamin, which are believed to contribute to its cardiovascular benefits [55,56,57]. A trial with sesamin, one of the lignans contained in sesame, is one of the few clinical studies using an isolated lignan compound rather than whole foods [58]. Results of this double-blind crossover trial showed intake of capsules with 60 mg sesamin per day for 4 weeks significantly reduced both SBP and DBP.

Epidemiological studies further support the association between lignan intake and blood pressure regulation. Data from the China Health and Nutrition Survey showed that higher dietary intake of polyphenols, particularly lignan and stilbene, was linked with a lower risk of hypertension [59]. Likewise, a study by Hu et al. [60], showed that individuals with greater lignan consumption exhibited a lower prevalence of hypertension and other cardiovascular risk factors. Moreover, a novel furofuran lignan, esquamosan, isolated from the leaves of *Annona squamosa* L., demonstrated a vasorelaxant effect in rats, primarily by inhibiting extracellular calcium influx, thereby providing pharmacological support for the plant’s traditional use in hypertension management [61].

**Figure 4:**
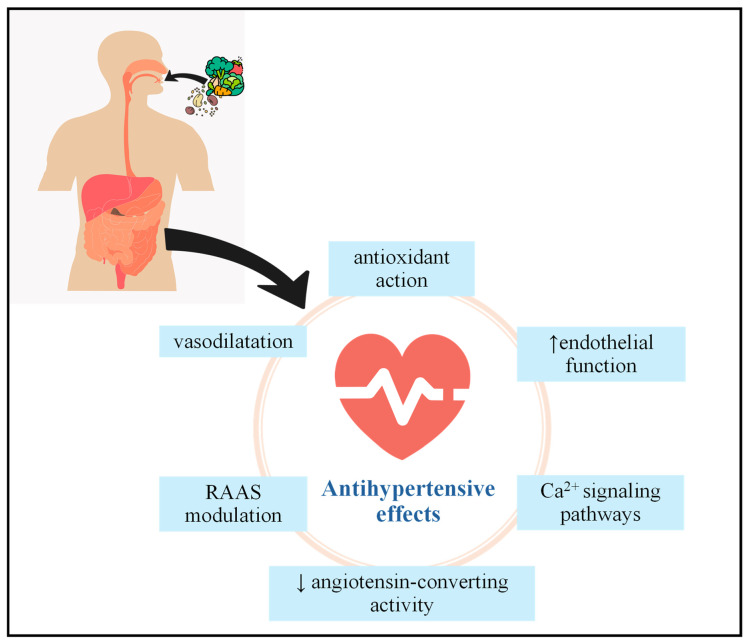
Main mechanisms involved in the antihypertensive effects of lignans.

The available evidence underscores the potential of lignans to moderately reduce blood pressure and improve vascular function. These effects are mediated through interacting molecular pathways, including enhancement of endothelial function, neutralization of ROS, inhibition of ACE activity, modulation of RAAS, and interaction with calcium signaling pathways (Figure 4). Together, these mechanisms provide a coherent biological basis linking plant lignan intake and microbiota-derived enterolignans to improved vascular health and reduced hypertension risk. The biotransformation of lignans by gut microbiota is central to their bioactivity, highlighting the importance of host–microbiome interactions in determining their antihypertensive efficacy.

### 4.2. Beneficial Role in Obesity

Obesity is a significant global health issue characterized by excessive accumulation of adipose tissue and chronic low-grade inflammation. According to the World Health Organization (WHO), individuals with a body mass index (BMI) of 30 or above are classified as obese, although specific criteria may differ between countries. As of 2022, approximately 43% of adults aged 18 years and older were overweight, with 16% classified as obese [62]. Obesity is primarily marked by hypertrophy and hyperplasia of adipocytes, abnormal lipid accumulation, systemic inflammation, insulin resistance, and dysbiosis of the gut microbiota [63]. These changes are strongly associated with various comorbidities, such as type 2 diabetes, hyperlipidemia, cardiovascular disease, and cancer [64]. The public health and socioeconomic burden of obesity underscores the need for effective and safe preventive and therapeutic strategies. Among emerging dietary interventions, lignans have shown promising potential in ameliorating obesity and its associated metabolic disturbances. Table 2 includes a compilation of selected studies reporting the anti-obesity effects of lignans on cell and animal models.

Experimental studies using rodent models, commonly fed high-fat diets (HFD) to induce obesity, have been instrumental in exploring the anti-obesity effects of lignans. HFD contributes significantly to hepatic steatosis and is a key driver of chronic diseases, for instance, nonalcoholic fatty liver disease (NAFLD), metabolic syndrome, and cardiovascular diseases [83]. Functional foods like flaxseed and sesame are rich in lignans and have been widely studied. In rats, supplementation with lignan-enriched flaxseed powder reduced body weight, adiposity, and blood pressure, while improving lipid profiles [45]. In mice, flaxseed lignans improved outcomes related to obesity-associated disorders primarily by enhancing insulin signaling and activating AMP-activated protein kinase (AMPK) [76]. Sesame oil, rich in lignans, has also demonstrated metabolic benefits. For example, Kim et al. [79], reported that sesame oil mitigated endoplasmic reticulum stress and apoptosis in the livers of HFD-fed mice. Moreover, sesaminol diglucoside stimulated thermogenesis in brown adipose tissue via beta 3 adrenergic receptors and protected mice against diet-induced obesity [77]. More recently, sesamol was shown to attenuate adipose tissue senescence in mice by scavenging reactive oxygen species and modulating the Nrf2/p38MAPK signaling pathway [82].

Obesity is an important risk factor for liver disorders, such as hepatic steatosis, dyslipidemia, hepatic insulin resistance, and NAFLD. Flaxseed lignans such as SDG were found to alleviate hepatic steatosis and insulin resistance in HFD-fed mice by improving lipid and glucose metabolism, primarily through AMPK activation [76]. Likewise, sesaminol demonstrated therapeutic potential against obesity-induced hepatic steatosis by modulating mitochondrial function, lipid metabolism, and inflammatory responses [80]. Schisanhenol, a biphenylcyclooctene-type lignan from the *Schisandraceae* family, was also shown to counteract NAFLD by enhancing lipolysis, promoting fatty acid oxidation, exerting anti-lipogenic effects, and activating AMPK-mediated pathways via inhibition of microRNA-802 [75].

Several lignans from other sources have also demonstrated anti-obesity potential. Arctiin, a lignan from *Arctium lappa* L. (burdock) was shown to reduce body weight and fat accumulation in HFD-fed mice as well as inhibit adipocytes by downregulating PPARγ and C/EBPα while activating AMPK [66]. Gomisin N, isolated from *Schisandra chinensis*, has been reported to reduce adipogenesis and prevent diet-induced obesity in mice [69]. Similarly, phillyrin, from *Forsythia suspensa* (Thunb.) was found to act through the PPAR-β/δ-ANGPTL4 axis to reduce body weight in obese models [84]. The gut microbiota is a critical factor in obesity development and energy homeostasis. Dysbiosis triggered by HFD alters host metabolism, promoting inflammation, lipid accumulation, and insulin resistance. Lignans can positively modulate gut microbiota composition and function, thereby influencing obesity-related pathways [63]. These compounds maintain microbial diversity and modulate levels of microbial metabolites involved in lipid synthesis and energy regulation. Upon lignans ingestion, lignans are metabolized by specific gut bacteria (*Bifidobacterium*, *Lactobacillus*, *Bacteroides*, and *Streptococcus*) into bioactive enterolignans [85]. For example, SECO, present in plants as a diglucoside, is converted into enterodiol and enterolactone by gut microbiota, with *Bifidobacterium* playing a central role in this transformation [63]. These microbial metabolites promote gut health by stimulating the growth of probiotic bacteria and enhancing the production of beneficial compounds, including short-chain fatty acids (SCFAs), vitamins, amino acids, and other essential nutrients [63,86,87]. Lignans may also function as prebiotics. For instance, arctigenin, a lignan from *Arctium lappa* L., demonstrated prebiotic properties by modulating gut microbiota and ameliorating obesity-associated dysbiosis, reducing inflammation, and restoring intestinal barrier function in obese mice [65].

Gut microbial composition is markedly altered in individuals with obesity, affecting energy harvest and leading to fat accumulation. Lignans have been shown to restore microbial balance, reduce fat synthesis, and inhibit adipose deposition [63]. Given the close link between lipid metabolism and obesity, lignans have also been studied for their lipid-lowering properties [88,89,90,91]. Experimental diets in male Sprague-Dawley rats supplemented with sesame lignans altered hepatic gene expression related to hepatic lipogenesis, cholesterogenesis, and glucose metabolism [92]. Sauchinone, a lignan derived from *Saururus chinensis*, has been shown to inhibit LXRα-mediated induction of SREBP-1c and its downstream effects, thereby preventing hepatic steatosis and protecting hepatocytes from fat-induced oxidative stress [72].

The gut–brain axis, which regulates appetite and satiety, is another key target for lignans. Intestinal dysbiosis can affect neuroendocrine signaling and food intake. Flaxseed supplementation, rich in lignans, has been shown to suppress appetite and enhance satiety [93,94]. Additionally, chronic low-grade inflammation, driven in part by lipopolysaccharide from Gram-negative gut bacteria, contributes to obesity. Increased lipopolysaccharide levels can trigger inflammatory cascades that begin in the gut and extend systemically [95]. Lignans may mitigate this inflammation by supporting beneficial microbiota and reducing intestinal permeability. Clinical studies reinforce these experimental findings (Table 3). Randomized controlled trials have shown that flaxseed products rich in lignans contribute to weight management by reducing appetite and promoting satiety [93,96,97]. In a double-blind, placebo-controlled trial involving hypercholesterolemic individuals, flaxseed lignan extract significantly decreased plasma cholesterol and glucose levels in a dose-dependent manner, correlating with the plasma lignan concentrations [91]. Another study in overweight and obese women showed that 12-week flaxseed supplementation improved adiponectin levels, potentially contributing to visceral fat reduction, although no significant changes were observed in serum lipid profiles [98]. Furthermore, population data from Dutch adults revealed an association between increased intake of lignan-rich plant-based foods and reduced waist circumference [99].

**Figure 5:**
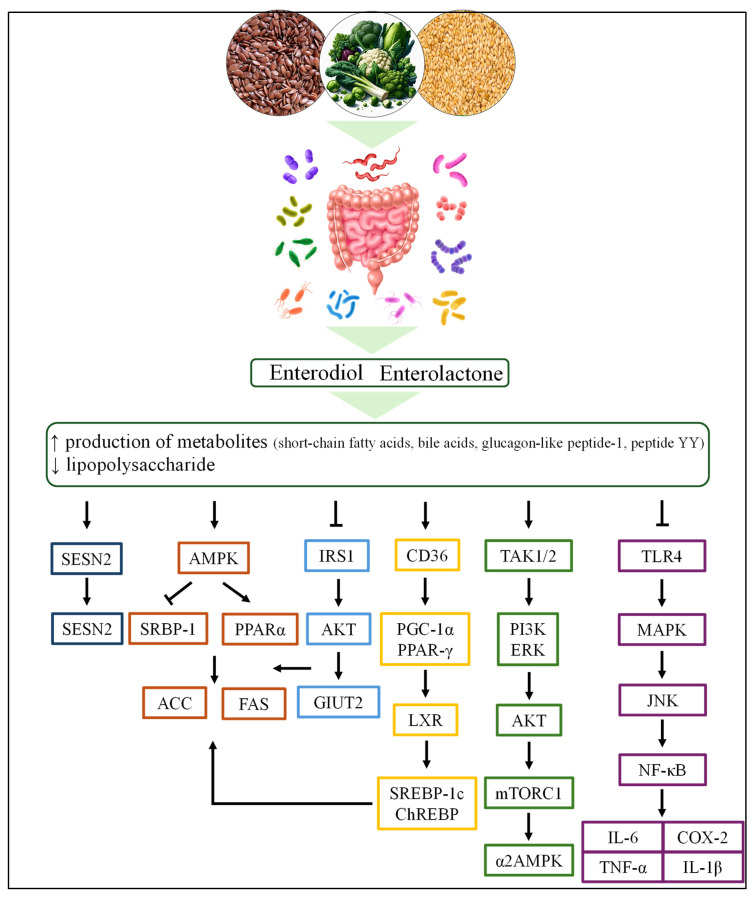
Main molecular mechanism of lignans against obesity involving different signaling pathways: AMPK/SREBP-1/PPARα/ACC/FAS and IRS1/AKT/GLUT2 are related to fat metabolism; TAK/PI3K/ERK/mTOR is involved in hypothalamic regulation of appetite; and TLR4/MAPK/p-JNK/NF-κB is related to inflammation. Adapted from Chu et al. [63].

Lignans exhibit promising anti-obesity effects through multiple interconnected mechanisms, including modulation of appetite-regulation hormones such as leptin and adiponectin, improvement of lipid metabolism, regulation of gut microbiota composition, enhancement of insulin sensitivity, reduction in inflammation, and attenuation of visceral fat accumulation. After ingestion, lignans are metabolized by the gut microbiota into enterodiol and enterolactone, which in turn promote the production of various metabolites—such as short-chain fatty acids (SCFAs), bile acids, glucagon-like peptide-1 (GLP-1), and peptide YY (PYY). These bioactive compounds act on multiple target organs and tissues through diverse signaling pathways (Figure 5), involving interconnected lipid metabolism, energy homeostasis, inflammatory signaling, adipocyte differentiation, and gut microbiome modulation. The microbial conversion of dietary lignans to enterolignans (enterodiol and enterolactone) reflects substantial differences in gut microbiome composition and functional capacity across individuals [104]. A subset of intestinal bacteria is associated with the ability to produce enterolignans, and people can be classified as high, low, or non-producers based on their microbial profiles and diversity. This is influenced by several factors, such as baseline diet (e.g., fiber intake), recent antibiotic or medication exposure, and broader microbiome ecology [105,106]. Generally, high diversity and the presence of specific taxa correlate with greater conversion efficiency, whereas disruptions such as antibiotics can diminish production. Because conversion efficiency can vary by several-fold even under similar lignan intake, circulating enterolignan levels not only reflect dietary exposure but also integrate microbial and host factors.

## 5. Extraction of Lignans

There is a pressing need to develop efficient, fast, and reliable technologies to achieve complete extraction and enrichment of lignans in samples for precise quantitative analysis. Nevertheless, the extraction of lignans can be harrowing due to these compounds’ structural complexity and diverse polarities [107]. The extraction of lignans from plant materials involves various strategies, including chemical processes traditionally used in the pulp and paper industry and techniques aimed at isolating specific bioactive compounds [108]. Among the chemical pulping methods, the Kraft process is the most widely used globally, accounting for around 85% of total lignin production [109]. This method operates under highly alkaline conditions, where approximately 90–95% of the lignin is solubilized into the resulting black liquor. Kraft lignin is typically recovered by acidifying the black liquor, commonly using sulfuric acid or carbon dioxide, followed by filtration, washing, and drying. Despite an annual production of more than 600,000 tons, most Kraft lignin is incinerated for energy generation, representing a low-value application [109].

On the other hand, the sulfite pulping process operates within a broader pH range (from 2 to 12), depending on the composition of the pulping liquor. This method yields lignosulfonates, a type of lignin-rich in sulfonate groups, which are widely used in industrial applications such as concrete additives, dispersing agents, and composite materials [110]. Meanwhile, the organosolv process has gained increased attention due to its sulfur-free approach. The organosolv method is a lignocellulosic biomass fractionation technique that uses organic solvents (such as ethanol, acetone, acetic acid, γ-valerolactone, among others), alone or mixed with water, to separate the main components of the biomass: cellulose, hemicellulose, and lignin. This method has been further promoted by the growth of the cellulosic ethanol industry, offering new opportunities for the valorization of lignin produced through this process.

**Figure 6:**
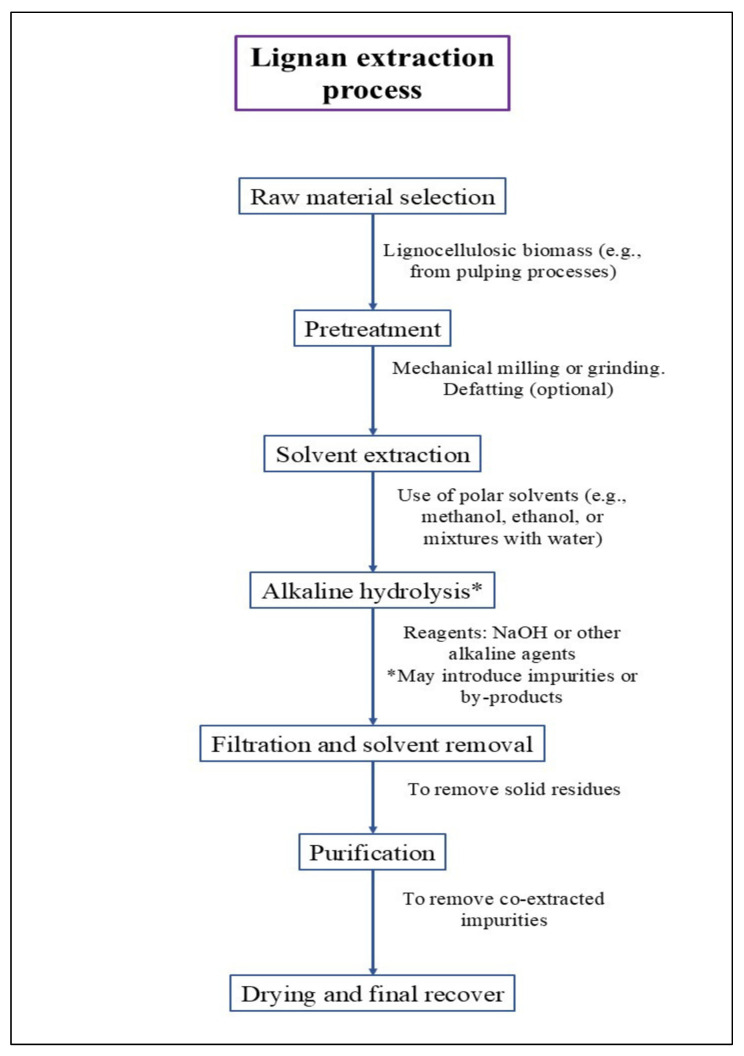
Process overview for solvent-based lignan extraction.

For the specific extraction of lignans such as SDG from flaxseed, polar organic solvents like methanol or ethanol, either pure or mixed with water, are commonly used to extract complexes formed with macromolecules such as 3-hydroxy-3-methylglutaric acid. The efficiency of this extraction varies depending on the solvent, so optimization methods like single-factor experiments, response surface methodology, and orthogonal design are frequently applied to improve results [111]. Following extraction, alkaline hydrolysis is typically used to release SDG. However, this step can introduce impurities or unintended by-products, which may lower the yield and enrichment quality (Figure 6). To address these limitations, environmentally friendly methods have become the preferred strategies for lignan recovery [112]. In this context, different technologies or methods have been used for lignan extraction from several food matrices, such as ultrasound-, microwave-, enzyme-assisted extraction, and subcritical fluid extraction, to mention a few (Table 4). The most direct comparison of the extraction technique yield efficiency is observed for sesame cake; under the evaluated conditions, accelerated-assisted solvent extraction provided the highest absolute recovery (9.34%), exceeding microwave-assisted extraction (5.75%) and ultrasound-assisted extraction (5.28%) by 62.4% and 76.9%, respectively. However, when efficiency is expressed as time-normalized throughput, microwave-assisted extraction exhibited the greatest productivity (1.15%·min^−1^) relative to ultrasound-assisted extraction (0.53%·min^−1^) and accelerated-assisted solvent extraction (0.47%·min^−1^), indicating that microwave heating can substantially reduce processing time at the expense of lower absolute recovery. For other matrices, ultrasound-assisted extraction showed high lignan recoveries in flaxseed (23.6 mg SDG/g) and *Asarum* sp. (asarinin 13.40 mg/g; sesamin 2.39 mg/g), while enzyme-assisted pretreatment coupled to subcritical extraction achieved 13.43 mg/100 g in sesame seed cake; nevertheless, these cross-study comparisons should be interpreted cautiously due to differences in substrate, target analyte expression, and reporting units.

Overall, these techniques enhance efficiency by reducing extraction time, lowering temperature requirements, and minimizing solvent use. For instance, ultrasound-assisted extraction improves mass transfer through mechanisms like cavitation and turbulence, and it can also enhance processes such as membrane separation, crystallization, adsorption, and aqueous two-phase extraction [111]. These methods significantly increase product recovery and processing speed at the industrial level, although additional purification steps are often necessary to remove co-extracted impurities before final analysis.

## 6. Development of Value-Added Products Containing Lignans

A wide range of value-added products enriched with lignans has been developed in recent years, including ground flaxseed and lignan-rich flax oil supplements, whole grain rye breads, flaxseed- or sesame-based snacks, and various plant-based functional foods. These are designed not only to enhance consumer appeal but also to provide added nutritional and therapeutic benefits. As per the data bridge market research, the global market size of lignans was valued at USD 496.42 million during 2025 and is expected to reach USD 859.35 million by the year 2033, which is mainly due to lignan consumption as cereal-based foods such as muesli, oatmeal, etc. (https://www.databridgemarketresearch.com/reports/global-lignans-market, accessed on 2 January 2026). Value-added food products are those whose market value is increased through processing, the incorporation of functional ingredients, or innovative packaging strategies. Such enhancements aim to improve sensory qualities, health benefits, or shelf-life, thereby increasing consumer acceptance compared to raw or unprocessed commodities [118]. In recent years, a number of studies on dietary intake of dietary lignans like phytoestrogens have been of specific interest with respect to hormone-related menopause, cancers, heart disease, and osteoporosis [119]. And development of more sensitive analytical techniques for the detection of lignan content in foods has allowed for the quantification of lignans in the commonly consumed foods in a more systematic way [120].

Flaxseed, recognized as one of the richest natural sources of lignans, has been widely used in the formulation of numerous value-added food products. These include flaxseed flour, roasted flaxseeds, flaxseed oil, flaxseed powder gum, flaxseed-enriched items such as chips, noodles, yogurt, and a diversity of baked goods including bagels, bread, muffins, cookies, pizzas, patties, biscuits, and buns [121,122,123]. Flaxseed oil is rich in α-linolenic acid and omega-3 fatty acids, and is well accepted by consumers. These two compounds are reported to have health-beneficial effects, such as lowering of bad cholesterol responsible for causing heart diseases, and some lignans can aid in reducing body weight and fat accumulation [45,124,125,126]. Incorporating flaxseed into wheat flour has been shown to enhance the nutritional profile of the final product by increasing mineral, lipid, protein, and dietary fiber content, while decreasing carbohydrate content [118]. Similarly, the addition of flaxseeds to baked and cereal-based products has been reported to improve both the functional and nutritional qualities of these foods, along with enhancing the amount of calories, protein, ash, acidity, and antioxidant properties [121,127]. The inclusion of flaxseed in bread formulations not only contributes to nutritional enhancement but also improves technological attributes such as water absorption, dough cohesiveness, and crumb softness [128]. Dairy products have likewise been successfully fortified with flaxseed oil and lignans, demonstrating the versatility of flaxseed as a functional ingredient across diverse food matrices [129]. Moreover, the development of ready-to-eat items, such as snacks, confectionery products, and crispbreads enriched with flaxseed, provides a convenient and appealing approach to increase consumer intake of lignans and their associated health benefits [119,120,122,130]. In addition, due to the high protein content in flaxseed, its byproduct can be used as a supplement in livestock, including poultry feed [131].

Lignans occur in low quantities in several plant species [132,133,134]. They possess numerous health-beneficial effects, and consumption of lignan-based crops has increased significantly during recent years [132,134]. However, the heredity-specific distribution and the lower production of lignans in natural plants have affected the effective and steady production of beneficial lignans [133]. In addition, the production of lignans from plants is declining day by due to suboptimal cultivation practices, high cost of extraction, low lignan content, and long generation cycles. Thus, research on making the advancement of modern technologies for improving lignan production has increased recently. The biotechnological approaches focus on alternative tools for the cultivation of value-added crops rich in lignans, because the plant in vitro cultivation has several benefits over collecting plants from fields [24,135,136,137]. Growing plant cells directly using modern technologies has advantages over the conventional methods since it provides a stringent control of the quality of the products without depending on the variations in natural production resulting from climate and socio-political changes in their countries of origin [138]. Current noticeable progress in the molecular and functional classification of lignan biosynthetic enzymes and endogenous and exogenous aspects for lignan biosynthesis has proposed new techniques for the metabolic engineering of lignan biosynthesis cascades that lead to the effective, maintainable, and steady lignan production in plants and their cell or organ culture [133]. There are several advances in lignan production which includes, molecular characterization of novel genes encoding enzymes for biosynthesis pathways of dietary and medicinal lignans; manufacture of both endogenous and exogenous lignans by transient or steady transfection of lignan biosynthetic genes into cultured cells, tissues and plants; and identification of exogenous stimuli such as light and elicitors that increase the production of lignans by cultured cells and plants [133]. Suspension cultures have attracted more interest due to their high growth rate and short cycle of reproduction, and the undifferentiated plant cells, maintained in a liquid medium, retain a high metabolic activity resulting in high yields in a short period of time [138,139]. Hairy root culture methods were also adopted for the production of high-yielding lignans [137]. In addition, authors have reported the use of in vitro micropropagation techniques to produce potent lignans and antioxidant secondary metabolites in linseed [140]. Others have used elicitors in the production of lignans [141,142]. Furthermore, several genetic engineering strategies have also been employed for enhanced production of lignan compounds [134,143]. Some authors have reported the use of RNAi-based metabolic engineering techniques to produce endogenous lignans [33,144]. By applying these modern technologies, the lignan contents in food crops can be enhanced. In summary, the development of value-added products containing lignans represents a practical and effective strategy to promote the incorporation of these bioactive compounds into the daily diet. Such functional foods not only meet consumer demand for healthier options but also support preventive health care through dietary intervention.

## 7. Safety, Limitations, and Translational Challenges

Laboratory research, when converted to clinical uses or when its application in the human treatment process is established, its value increases. However, a lot of limitations and translational challenges are faced by lab research to convert completely to value-added drugs passing through a series of clinical trials. Lignans are structurally and functionally varied phytochemicals found in diverse plant species (flaxseed, beans, fruits, vegetables, and some whole grains) that have received wide consideration as leading compounds for healthy diets to reduce the risks of lifestyle-related non-communicable diseases [133]. Lignans are phytoestrogens that possibly influence human health and have proven effective against a number of diseases, such as cardiovascular disease and obesity [145,146,147]. Consequently, the formulation of new technology for the mass production of lignan is a recent interest among scientists. Recent advances in the molecular and functional classification of lignan biosynthetic enzymes and endogenous and exogenous factors for its biosynthesis have proposed several approaches for the metabolic engineering of lignan biosynthesis cascades, resulting in an effective, viable, and established lignan production in the plants, together with plant cell/organ cultures [133]. As they are present in a wide variety of foods, nutritional lignans are commonly measured to be safe with no harmful side effects when consumed [146]. However, certain caution should be taken in premenopausal women and newborns when it comes to timing and method of administration of the dose, as unsuitable estrogenic action might cause unwanted side effects [148].

Lignans, like other polyphenols, face various challenges in drug development due to their properties such as low and variable oral bioavailability, low water solubility, chemically unstable nature, variability in extraction and quantification methods, suspicions about long-term safety, and an absence of vigorous clinical evidence from human trials [2,145,149]. Poor bioavailability of lignans restricts their solubility in water, making their absorption in the gastrointestinal tract inefficient [150]. In addition, the lignin’s polarity also affects its extraction efficiency, and more inorganic solvents are required for the extraction of its promising bioactive compounds [2]. Processing has a substantial effect on the bioaccessibility of bioactive compounds, and presently, there are very few reports on the effect of various processing methods on the bioaccessibility of lignans in lignan-rich food, in comparison to other studies on polyphenols [151]. A study reported that the presence of bile salt in the intestine could have affected the bioaccessibility since it reduces the solubility of sterols [152,153]. And the process of germination and fermentation could improve the lignan bioaccessibility [153]. In addition, despite its rich beneficial properties, there is little data available on the bioaccessibility of lignans, both in isolated systems and in food matrices [2]. Lignans frequently undergo considerable metabolic failure in the liver before passing into the systemic circulation, thereby disturbing their therapeutic proficiency [149]. Some lignan compounds undergo first-pass metabolism, resulting in less systemic bioavailability [149]. Some studies have reported the effect of flaxseed on blood pressure, cholesterol level, and body mass index; however, it is not sufficient, and further clinical research on different lignan sources and the molecular mechanism of action is needed since different forms of flaxseeds may have different effects [154]. Though a series of animal studies on lignans have been carried out, the exact mechanism of action and the specific lignan compounds responsible for these activities are still not clear [28]. In addition, direct comparison between animal study and human trials with respect to doses needs to be extensively studied [28].

Extraction and quantification of lignans from plant sources can also be challenging sometimes. Furthermore, variability in the extraction and purification methods is vital to attaining reliable lignan composition and concentrations, safeguarding reproducibility, quality, and effectiveness in the beneficial applications [28,149]. Most of the clinical trials conducted are observational only, have been of comparatively short periods, and have involved a small number of patients. Further studies on the potential adverse effects of long-term use of lignans in humans and clinical effectiveness are also essential before their potential application. The bioactivity potential of plant lignans is generally connected with their alternation by the gut microbiota into enterolignan compounds (enterolactone and enterodiol), and since the gut microflora is not the same for all humans, the efficiency of the lignan-based drugs will vary accordingly [155]. Furthermore, other factors like type of diet, food transit time, and the intestinal redox state can also control the lignans bio-activation by the human gut microbiota [155].

In spite of the interesting bioactive potential and preclinical efficacy of lignans, various translational challenges and hurdles need to be addressed before advocating their applications toward their clinical potentials [156]. First, the pharmacological dominance of the naturally occurring lignan over its isolates still needs to be proven [156]. Secondly, its structure-activity relationship also needs to be addressed. Factors such as the gut microbiota composition and the genetic variability of individuals also affect the nature of the different metabolites or compounds absorbed into the systemic circulation and thus complicate the pharmacokinetic profiling, a crucial step towards drug development [157,158]. In addition, another issue is the bioavailability of lignans in humans, and reports suggest that there is a lack of relationship between the intake of lignans and their distribution in tissues [159]. This inconsistency hampers the transformation of in vitro findings to in vivo models, or the translation of the preclinical studies into clinical applications, affecting the development of lab research to therapeutics. This knowledge gap hampers balanced drug design efforts to improve lignan effectiveness or reduce off-target effects [156]. Hence, the future research should focus on various interconnected aspects to bridge these knowledge gaps. In addition, the optimization of both mechanistic and pharmaceutical approaches should be carried out, which will help explain the detailed molecular originators of lignan action, elucidate crosstalk between regulated pathways, and loosen the basis for model-dependent inconsistency in effectiveness [156]. Clinical translation and authentication must be prioritized, with a focus on biomarker-guided, randomized controlled trials, dosage form optimization, chemical alterations, and additives to improve bioavailability and targeted delivery that utilize well-standardized lignan extracts/ compounds [145]. Advances in biomaterials and encapsulation research have shown some promising potential in addressing the bioavailability issues of these compounds, paving the way for more effective therapeutic applications. With respect to their diverse beneficial effects, lignans signify an imperative and growing area of interest among food as well as pharmaceutical scientists. However, in spite of considerable proof from in vitro and animal studies, its clinical data remain inadequate. Further human trials are essential to find out the ideal dose, develop an understanding of its bioavailability, and establish its beneficial effectiveness. More detailed research on the age, sex, and gut microbiota composition and their effect on the lignan metabolism and activity needs to be studied.

## 8. Conclusions and Future Prospects

Lignans, mainly found in dietary sources (seeds, whole grains, vegetables, and fruits), are some of the most relevant biomacromolecules with a wide range of benefits for human health, such as anti-inflammatory, anticancer, alleviating menopausal symptoms, reducing the risk of type 2 diabetes, and protecting cardiovascular health, among others. Scientific evidence from animal and clinical trials reviewed suggested that lignans also provide important beneficial effects in lowering blood pressure and in reducing body weight, BMI, and central adiposity. Lignans can reduce blood pressure and improve vascular function, which is mediated through multiple mechanisms, such as enhancement of endothelial function, neutralization of ROS, inhibition of ACE activity, modulation of RAAS, and interaction with calcium-signaling pathways. Furthermore, the anti-obesity effects of lignans are mediated through multiple interconnected mechanisms, such as modulation of appetite-regulation hormones such as leptin and adiponectin, improvement of lipid metabolism, improvement of insulin sensitivity, reduction in inflammation, attenuation of visceral fat accumulation, and particularly by regulation of gut microbiota composition. Lignans are not directly metabolized by digestive enzymes in the human gastrointestinal tract, after intake dietary lignans are converted by gut microbiota into bioactive enterolignans, the main physiologically active forms of lignans that provide the biological effects and promote the production of metabolites (e.g., such as SCFAs, bile acids, GLP-1 and PYY, etc.) that act on different target organs or tissues through different pathways. Hence, the gut microbiota plays a key role in revealing the bioavailability and functionality of lignans, highlighting the importance of host–microbiome interactions in determining their antihypertensive and anti-obesity efficacy. Nevertheless, it will be important to evaluate the changes in metabolites of intestinal contents and clarify the way these metabolites influence the liver fat metabolism by the gut–liver axis. Moreover, the target genes and signaling pathways involved in these processes should be clarified in future studies to fully elucidate the molecular mechanisms involved. Additionally, the investigation of possible synergistic effects with other bioactive compounds and phytochemicals could improve their clinical usefulness. In addition, formulation of value-added lignan-based functional foods or supplements with increased bioavailability could also facilitate its potential application in the food industry. Collectively, the reviewed findings support the potential of lignans as valuable dietary components in lowering blood pressure and in ameliorating obesity and associated metabolic disorders, although definitive proof of direct effects of isolated lignans in humans remains limited.

## Figures and Tables

**Table 1:** Some reports on the antihypertensive effects of lignans on animal models.

Product/Compound	Model	Dose/Duration of Treatment	Findings	Reference
Flax lignan concentrate (FLC)	Hypertensive rats	FLC (200, 400, and 800 mg/kg, p.o.) was administered daily to the rats for 5 weeks.	FLC (400 and 800 mg/kg) significantly reduced the systolic blood pressure and diastolic blood pressure via modulation of endogenous enzymes.	[44]
Gomisin J from *Schisandra* *chinensis*	Hypertensive mice	Mice were treated with 1 or 3 μg/kg/min gomisin J for 14 days.	Gomisin J reduced the rise in arterial blood pressure by preserving vascular nitric oxide (NO) bioavailability, inhibiting reactive oxygen species (ROS) production, and preventing the dysfunction of endothelial nitric oxide synthase (eNOS).	[48]
Phillygenin	Hypertensive rats	Rats were orally treated with 2.5–10 mg/kg phillygenin	Phillygenin reduces hypertension by reducing PLCβ3-dependent Ca^2+^ oscillation.	[49]
Secoisolariciresinol diglucoside (SDG) from flaxseed	Sprague Dawley male rats	Rats were treated with 10 mg/kg SDG.	SDG attenuated the angiotensin I-induced increases in systolic, diastolic, and mean arterial blood pressures.	[46]
Secoisolariciresinol diglucoside lignan-enriched flaxseed powder (LEFP)	Rats fed a high-fat and high-fructose diet	The diet was added with 0.02% LEFP for 12 weeks.	LEFP lowered blood pressure.	[45]

**Table 2:** Some reports on the anti-obesity effects of lignans on cell and animal models.

Product/Compound	Model	Dose/Duration of Treatment	Findings	Reference
Arctigenin	HFD mice	Mice were treated with 100 mg/kg arctigenin	Arctigenin improves metabolic disorders by reshaping the gut microbiota and regulating the GPR/HDAC3 and TLR4/NF-κB signaling pathways.	[65]
Arctiin	3T3-L1 cells and HFD mice	3T3-L1 cells were treated with arctiin at concentrations ranging from 12.5 to 100 μM for 8 days, while rats received a daily dose of 500 mg/kg arctiin for 4 weeks.	Arctiin inhibited adipogenesis by suppressing PPARγ and C/EBPα expression and activating the AMPK signaling pathway.	[66]
Deoxyschizandrin	HFD obese mice	Obese mice were fed either a lignan-free diet or a diet supplemented with 65 mg/kg/day of lignans for 6 weeks.	Deoxyschizandrin alleviates obesity by regulating the activity of the farnesoid X receptor, bile acid receptor 1, and leptin signaling pathways.	[67]
*Fructus arctii*	KKAy mice	KKAy mice were fed either a lignan-free diet or a diet supplemented with 125 or 250 mg/kg/day of *Fructus arctii* for 11 weeks.	*Fructus arctii* reduced body weight, serum triglycerides, and free fatty acid levels in the mice.	[68]
Gomisin N	3T3-L1 preadipocytes and HFD mice	Preadipocytes were incubated with 10–100 µM Gomisin N; mice were orally treated with 2 or 10 mg/kg Gomisin N.	Gomisin N repressed the differentiation of 3T3-L1 preadipocytes, reduced body weight gain, fat pad weight, adipocyte sizes, serum levels of glucose, triglyceride, as well as hepatic triglyceride in the HFD-induced obese mice.	[69]
*Litchi chinensis* seed	HFD zebrafish and mice	HFD zebrafish were treated with a lignan-free diet or a diet containing 0.35 or 1.4 mg/d lignan for 8 weeks; HFD mice were treated with either a lignan-free diet and a diet containing 300 or 500 mg/d lignans for 12 weeks.	*Litchi chinensis* seed can diminish the weight of HFD zebrafish and ice, improve lipid accumulation and lipid metabolism, control appetite, and prevent hepatoenteric inflammation.	[70]
Phyllanthin	HFD mice	HFD mice were treated with either a lignan-free diet or a diet with phyllanthin (2 or 4 mg/d) for 12 weeks.	Phyllanthin consumption can ameliorate the development of metabolic disorders.	[71]
Sauchinone	HFD mice	HFD mice were treated with either a lignan-free diet or a diet with sauchinone (10 or 30 mg/kg) for 11 weeks.	Sauchinone inhibits hepatic steatosis, protecting hepatocytes from oxidative stress caused by fat accumulation.	[72]
*Schisandrae chinensis* oil	Diabetic Wistar rats	Oil (1 mg/kg) was administered orally by gavage daily for 8 weeks to diabetic and normal rats.	The oil enhances pancreatic β-cell function by boosting the antioxidant capacity of the pancreas and increasing the expression of genes involved in glucose metabolism.	[73]
Schisandrin	Depressive mouse model induced by lipopolysaccharide.	Mice were fed either a lignan-free diet or a diet supplemented with lignans (30 mg/kg/day) for 14 days.	Schisandrin restored intestinal microbial balance in inflammatory mice by inhibiting the expression of the TLR4/NF-κB signaling pathway.	[74]
Schisanhenol	Liver disease mouse model induced by HFD non-alcoholic fatty.	Mice were treated with 5, 10, or 20 mg/kg schisanhenol.	Schisanhenol improved lipolysis and fatty acid oxidation, showed anti-lipogenic activity, and modulated AMPK-mediated lipid metabolism by inhibiting miR-802.	[75]
Secoisolariciresinol diglucoside (SDG)	HFD mice	SDG (0.05%, *w*/*w*) supplementation for 16 weeks.	SDG mitigates hepatic steatosis and improves insulin resistance.	[76]
Secoisolariciresinol diglucoside lignan-enriched flaxseed powder (LEFP)	HFD rats	Normal and HFD rats were treated with 0.02% LEFP	LEFP reduced body weight and fat accumulation, improved lipid profiles, and helped regulate blood pressure.	[45]
Sesame extracts and sesaminol diglucoside	HFD mice	Mice were treated with 20 mg/kg sesame extract and 5 mg/kg sesaminol diglucoside.	Sesaminol diglucoside induced brown adipose tissue thermogenesis through β3-AR.	[77]
Sesame lignans (sesamin and sesamolin)	Rats	A diet containing either 0.6 or 2 g/kg of lignans (sesamin or sesamolin), or a combination diet with sesamin (1.4 g/kg) and sesamolin (0.6 g/kg), was administered for 10 days.	Sesamin and sesamolin increased both the activity and mRNA expression of several enzymes involved in hepatic fatty acid oxidation and lipogenesis.	[78]
Sesame oil lignans	HFD mice	Mice were treated with sesame oil for 12 weeks.	Sesame oil reduced endoplasmic reticulum stress and apoptosis.	[79]
Sesaminol	Liver disease in a mouse model induced by HFD non-alcoholic fatty.	Mice were treated with sesaminol (2 mg/kg).	Sesaminol influences mitochondrial function, lipid metabolism, and inflammation.	[80]
Sesamol	Mice	Wildtype and ApoE^−^^/−^ mice were treated with an HFD and sesamol (0.05%, *w*/*v*, in drinking water) for 10 weeks.	Sesamol can restructure the intestinal environment of HFD mice and boost the production of SCFAs.	[81]
Sesamol	Aged obese (HFD) mice and a senescent cell model.	Mice were treated with 100 mg/kg sesamol for 8 weeks.	Sesamol ameliorates oxidative stress-intensified adipose tissue senescence in aged obese mice through Nrf2/p38MAPK signaling.	[82]

HFD: high-fat diet; SCFAs: short-chain fatty acids.

**Table 3:** Anti-obesity effects of lignans in clinical studies.

Population	Product/Compound	Dose and Duration	Key Outcomes	Reference
Overweight/obese women	Milled flaxseed	30 g/day, 12 weeks	↓ waist circumference, ↓ waist-to-hip ratio; ↑ adiponectin	[95]
Adults with overweight/obesity	Flaxseed/flax products (whole, lignan, oil)	varied; ~≥10–20 weeks	↓ body weight, ↓ BMI, ↓ waist circumference	[100]
Adults with overweight/obesity	Flaxseed	varied	↓ body weight, ↓ BMI, ↓ waist circumference	[101]
Type 2 diabetics	Ground flaxseed vs. wheat bran	40 g/day, 12 weeks	Did not show significant weight/BMI change; improved metabolic markers	[102]
Older obese adults	Flaxseed lignan complex vs. placebo (with exercise program)	~543 mg/day, 6 months	Smaller waist circumference gain (central adiposity trend)	[103]
Children and adolescents with overweight/obesity	Flaxseed or puffed wheat	20 g/d flaxseed or 25 g/d puffed wheat for 4 weeks.	↓ BMI, ↓ appetite, and waist circumference.	[65]

**Table 4:** Parameters and extraction methods of lignans from several sources.

Source	Extraction Method	Conditions	Yield or Lignan Content	Reference
*Asarum* sp.	Ultrasound-assisted extraction	S: 80% ethanol, t: 40 min, T: 51 °C, SS: 1 g/10 mL	13.40 mg asarinin/g/g and 2.39 mg sesamin/g/g	[113]
Flaxseed	Ultrasound-assisted extraction	S: Water supplemented with 0.2 N NaOH, t: 60 min, T: 25 °C, SS: 50 mg/10 mL	23.6 mg SDG/g	[114]
Flaxseed cake	Microwave-assisted extraction	S: 70% Methanol with 1 M NaOH, t: 3 min, SS: 500 mg/20 mL	16.1 mg SDG/g	[115]
Oat powder (*Avena sativa* L.)	Ultrasound-assisted extraction	S: 84% methanol, t: 60 min, T: 40 °C, SW: 0.1 g	59.56 µg/100 g	[116]
Sesame cake	Ultrasound-assisted extraction	S: 71% ethanol, t: 10 min, T: 50 °C, SS: 1.50 g/20 mL	8.28%	[117]
	Microwave-assisted extraction	S: 80% ethanol, t: 5 min, T: 50 °C, SS: 0.85 g/20 mL	8.75%	
	Accelerated-assisted solvent extraction	S: 80% ethanol, t: 20 min, T: 65 °C, SW: 2 g	9.34%	
Sesame seed cake	enzyme-assisted treatment + subcritical fluid extraction	Enzyme assisted E: cellulase + pectinase+ xylanase + β-glucosidase, SW: 6 g Subcritical extraction S: 1000 mL dimethyl ether, P: −0.1 MPa, T: 40 °C, t: 30 min, SW: 200 g	13.43 mg/100 g	[15]

E: Enzyme, t: time, T: Temperature, P: Pressure, S: Solvent, SS: solid-to-solvent, SW: solid weight.

## Data Availability

No new data were created or analyzed in this study.
